# The Effect of a Pace Training Session on Internal Load and Neuromuscular Parameters in Taekwondo Athletes

**DOI:** 10.3389/fphys.2021.710627

**Published:** 2021-08-03

**Authors:** Jader Sant' Ana, Raphael Luiz Sakugawa, Fernando Diefenthaeler

**Affiliations:** Laboratório de Biomecânica, Departamento de Educação Física, Universidade Federal de Santa Catarina, Florianópolis, Brazil

**Keywords:** martial arts, heart rate deflection point, physical preparation, mobile technology, anaerobic threshold

## Abstract

This study aimed to verify the effect of a pace training session at an intensity corresponding to the kick frequency at the anaerobic threshold (KF_AT_) on the internal load response and motor response performance of the roundhouse kick. Twelve black belt taekwondo athletes underwent two evaluation sessions: (1) performed the progressive specific test for taekwondo (PSTT) to identify the heart rate deflection point (HRDP) and the KF_AT_; (2) performed three 2-min rounds with a 1-min interval. Heart rate (HR) throughout each round and motor response performance before and after sessions were measured. The Student's *T*-test or Wilcoxon test was used, and *p* < 0.05 was adopted. During round 1, a lower internal load was observed (167 ± 10 bpm) compared with HRDP (179 ± 8 bpm; *p* = 0.035). During rounds 2 (178 ± 10 bpm; *p* = 0.745) and 3 (179 ± 8 bpm; *p* = 1), no differences were observed for an internal load and HRDP. Motor response performance showed no differences. However, a potentiation in the post countermovement jump test compared with rounds 1 (*p* = 0.012) and 2 (*p* = 0.028) was observed. The internal load (HR) observed at the intensity corresponding to KF_AT_ can be considered in the prescription of training when the aim is to control the internal load responses without inducing fatigue.

## Introduction

Taekwondo is characterized as an intermittent sport, with alternation between attacks involving high-intensity movements and periods of low intensity, or even periods of inactivity (Matsushigue et al., 2009). These characteristics are reflected in an effort: pause ratio (E:P) during combat of from 1:4 to 1:9 (Matsushigue et al., 2009; Santos et al., 2011; Campos et al., 2012; Del Vecchio et al., 2016). During international-level taekwondo competitions, athletes perform 8 ± 3 high-intensity attacks, lasting around 1.3 ± 0.4 s each, combined with 9.2 ± 3.9 s of bouncing movements and 6. ± 3.9 s of referee interruptions, resulting in 1:9 attack to the stepping movement ratio and 1:15 high-intensity actions to the low-intensity and pause ratio (Santos et al., 2011).

Thus, the motor actions in taekwondo that are characteristics of the modality, which imposes the E:P relationship, generate energetic alternations between the moments of aerobic predominance and those determining moments when the anaerobic demand increases (Campos et al., 2012). It is important to note that success in sports such as taekwondo is often associated with rapid motor actions in response to a particular stimulus (Bouhlel et al., 2006; Loturco et al., 2017), such as, for example, the reaction and response times of the kicks (Vieten et al., 2007; Hermann et al., 2008).

In combat sports, the determinant actions that involve anaerobic power (i.e., kick) are supplied by anaerobic energy systems, in particular the anaerobic alactic system (ATP-CP) (Bridge et al., 2009; Obmiński et al., 2011). These actions demand high production of force in small-time intervals, which is known as “the rate of force development” (Lars et al., 2006), and promote increased recruitment of muscle fibers, especially those with rapid contraction (Maffiuletti et al., 2016). Taekwondo athletes must maintain the ability to sustain decisive actions, high anaerobic power, and neuromuscular performance throughout the fight, without compromising the premotor reaction time and response time of the muscles associated with the kick (Sant' Ana et al., 2017).

Therefore, the evaluation of muscle power in combat sports athletes is important. However, although the use of vertical jump tests for this purpose has been questioned (Morin et al., 2019), the countermovement jump test (CMJ) is often used (Loturco et al., 2017). In addition, there is evidence of strong correlations between mean power in the CMJ test and shorter kick cycle times (*r* = −0.89) and mean kick cycle times (*r* = −0.79) in a specific test to assess the power and anaerobic capacity of taekwondo athletes (Sant' Ana et al., 2014). The CMJ test has also been able to discriminate the competitive level of combat sports athletes (Ravie et al., 2004).

Furthermore, the determinant high-intensity actions associated with predominant low-intensity actions, which reflect the external load (i.e., pace or E:P ratio), and consequently generate physiological responses (an internal load) (Kirk et al., 2015), need to be controlled. Some studies show different values of external load (ratio E:P) and internal load responses (e.g., blood lactate and heart rate—HR) in simulated combat situations (Campos et al., 2012), simulated competitions (Bouhlel et al., 2006; Butios and Tasika, 2007), small combat games with each athlete confronting one or two opponents in different area sizes (i.e., 4 × 4 m, 6 × 6 m, and 8 × 8 m) and imposed ratio E:P-−1:2 or free combat (Ouergui et al., 2020, 2021), competitions with a single round (Matsushigue et al., 2009), and official competitions (Bridge et al., 2009; Obmiński et al., 2011). These observational studies, based on means, make it difficult to understand the relationship of external and internal load parameters individually, and the effects of these on neuromuscular performance and determinant actions (kicks) performed by the athlete during combat.

Currently, in taekwondo, the internal load (e.g., HR) has been estimated from external load parameters (e.g., kick frequency—KF) or the pace obtained during progressive specific taekwondo test (PSTT) (Sant' Ana et al., 2019). During these tests, it is possible to obtain an individualized kick frequency at the anaerobic threshold (KF_AT_) pace or frequency that can be used to modulate the intensity of the training sessions and, also, to verify the effects on specific parameters through the relative intensity of the test (Sant' Ana et al., 2017). However, the literature lacks studies that have verified the effect of using the specific external load (i.e., KF_AT_), identified in PSTT, on the internal load response, neuromuscular performance, and the specific technique of taekwondo athletes (e.g., kick). Thus, the present study aimed to verify the effect of a pace training session performed at the intensity corresponding to KF_AT_ on the internal load response, performance during the CMJ test, and parameters associated with the neuromuscular and motor performance of the roundhouse kick through the evaluation of premotor reaction time, motor reaction time, and kick response time in taekwondo athletes. Our first hypothesis was that pace training at an intensity corresponding to KF_AT_ would promote internal load responses similar to those observed at the pace intensity determined by the HRDP during the incremental test. Our second hypothesis was that three rounds at a pace-related intensity would cause a drop in CMJ performance and worsen the parameters associated with the motor response time of the roundhouse kick of taekwondo athletes.

## Materials and Methods

### Participants

The sample was an intentional non-probabilistic type composed of a group of 12 male black belt taekwondo athletes (20.7 ± 4. years; 177 ± 6 cm; 73.6 ± 7.9 kg, 12.1 ± 3.5% body fat, and 8.4 ± 5. years of practice). The athletes participating in the study were engaged in at least 1 h of training three times a week. The athletes regularly participated in regional and state competitions, and some of the athletes (three athletes) were national and international level competitors. Considering the Olympic classification of weight division, the participants were referred to as flyweight (one athlete), featherweight (five athletes), welterweight (four athletes), and heavyweight (two athletes).

### Study Design

**Figure 1:**
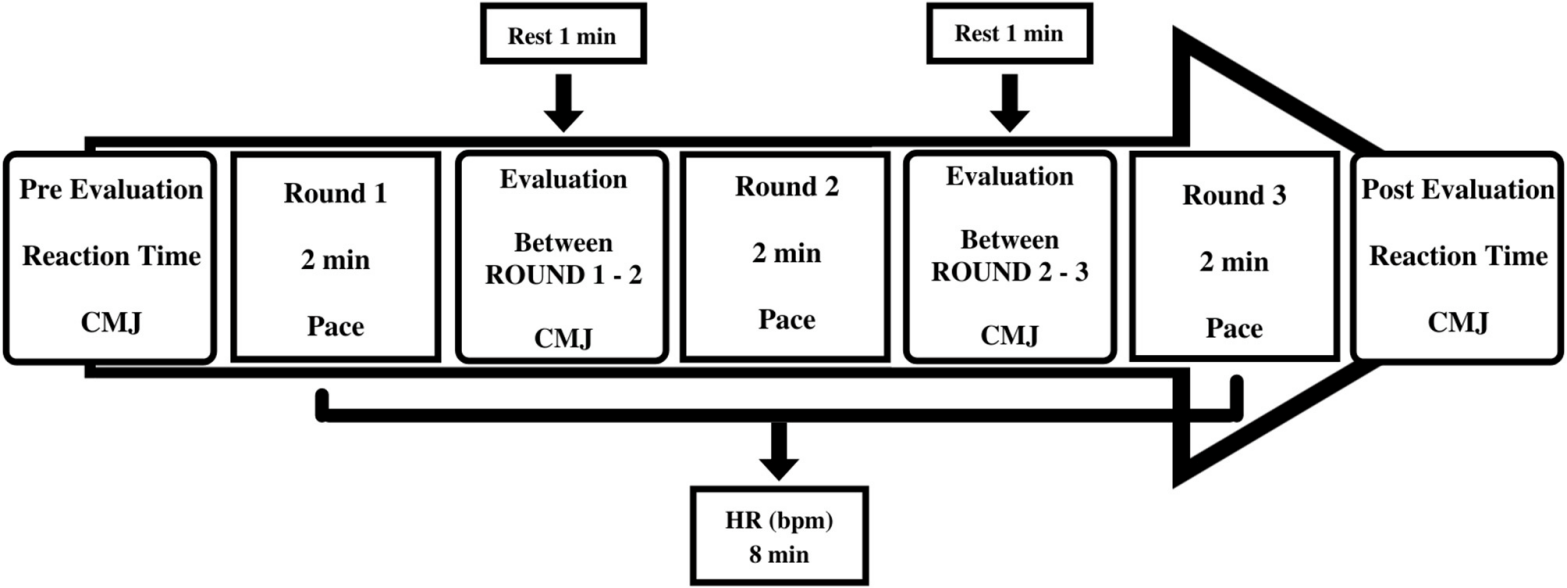
Experimental design of evaluation protocol before, during, and after pace training sessions.

Data collection was carried out in two sessions in the following order: (1) PSTT and (2) training at pace-related intensity. The interval between each session was longer than 48 h. In the first session, the athletes performed the PSTT to identify the HRDP, KF_AT_, and the intensity related to the training pace through the mobile application *ITStriker* (ETS4ME, São José, SC, Brazil). In the second session, the athletes performed three rounds of 2 min with a 1-min interval, with HR being measured throughout each round, CMJ jump performance and variables associated with premotor reaction time, motor reaction time, and time response of the roundhouse kick pre and post the pace training sessions in the intensity of KF_AT_ (Figure 1). The athletes were instructed not to perform any other type of physical effort in the 24 h before data collection. Each individual was informed about the risks and benefits associated with the test protocol. All research procedures were previously approved by the Ethics and Research Committee on Human Beings (protocol No. 145882).

### Procedures

#### Anthropometric Evaluation

To characterize the group of athletes, the following anthropometric variables were measured: height using a stadiometer (Sanny, São Paulo, Brazil), body mass with an electronic scale (Toledo, São Paulo, Brazil), with 0.1-cm and 0.1-kg resolutions, respectively, and thickness of the skin folds was measured, using a scientific compass (Cescorf, Porto Alegre, Brazil), with a 0.1-mm resolution. The percentage of fat was calculated from the Siri equation (Siri, 1961), using the body density established for men (Jackson and Pollock, 1978) obtained through the following skin folds: subscapular, midaxillary, triceps, thigh, supra-iliac, abdomen, and chest. Three measurements were made at each point, all on the right side of the body, and the mean value or value that was repeated two times was recorded. All measurements were performed by a single experienced evaluator.

#### Progressive Specific Test for Taekwondo Practitioners—PSTT

To perform the PSTT and identify the maximum kick frequency (KF_MAX_), maximum heart rate (HR_MAX_), HRDP, KF_AT_, and training pace by the Dmax method (Kara et al., 1996), the *ITStriker* application and a Bluetooth belt were used to record the HR (Polar H7® Kempele, Finland), paired with the application. The PSTT was performed in an area of 2 × 2 m demarcated by a mat. A “punching” bag was used for the kicks, which were required to be carried out at a height between the umbilical scar and nipples of the athlete. The subjects began the PSTT with the right leg, and the first stage started with a frequency of six kicks, alternating the legs, with an increase of four kicks at each new stage. During the test, the athletes always kept in step (jumping position) and performed the protocol until exhaustion. For more information about the test, see (Sant' Ana et al., 2019).

#### Protocol for Pace Training Session

Before trials, the athletes performed a 5-min warm-up, consisting of stretching step movements, knee lifts, and 10 roundhouse kicks performed with each leg. The pace training protocol started 3 min after the warm-up, and the athletes performed three 2-min rounds with a 1-min interval, and HR was measured throughout each round, using a Bluetooth belt paired with the *ITStriker* application. For maintenance of the KF_AT_ and the training pace corresponding to that obtained in the PSTT, the *ITStriker* application was used in the training mode to ensure that the individual pace intensity related to the HRDP of each athlete was maintained throughout all rounds. In addition, to reproduce the technical-tactical actions closer to those carried out in combat, kicks were used with variations in the techniques and combining up to three kicks for each pace, always alternating steps, hoopings, and movements characteristics of taekwondo.

#### Determination of Premotor Reaction Time, Motor Reaction Time, and Response Time

**Figure 2:**
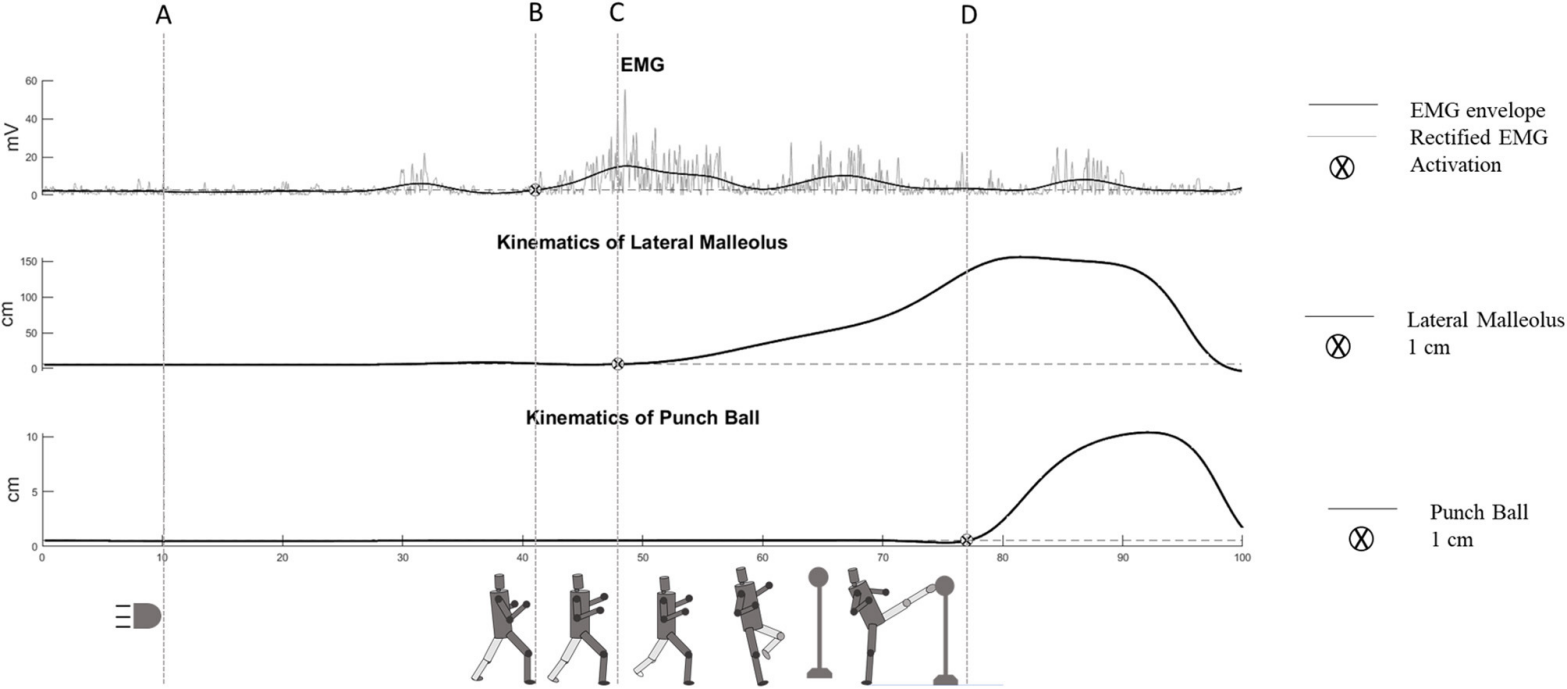
Measurement of the parameters associated with the neuromuscular and motor performance of the roundhouse kick from a representative participant. The dot line **(A)** represents the visual stimulus; **(B)** represents the electromyography activation onset of the rectus femoris muscle; **(C)** represents the kinematics of the lateral malleolus onset (movement of 1 cm of the lateral malleolus marker), and **(D)** represents the target onset (movement of 1 cm of the punch ball marker).

To determine the premotor reaction time and the response time, synchronization was performed between a light signal and the electromyography (Delsys, Inc., Natick, MA, USA) and kinematics system (Vicon, Oxford Metrics, Oxford, UK). The motor reaction time was determined with kinematics, using reflective markers on the joint of interest. The athletes wore a sock with reflective markers (ankle and lateral malleolus). All the athletes performed three kicks pre and two kicks post the pace protocol, and the best pre and post kicks were used for analysis (Figure 2).

To identify the premotor reaction time, defined as the time interval between the visual stimulus and the electromyography activation onset, the activation signal of the rectus femoris muscle generated in each executed kick was identified. As a criterion for determining the premotor reaction time based on muscle activation, using surface electromyography (EMG), the signals were amplified and recorded at a sampling rate of 2,000 Hz, and the raw EMG signals were smoothed with a fourth order Butterworth digital recursive filter (20–500 Hz). Baseline EMG activity was assessed 150 ms before each light signal used to determine the kick execution. An increase in the EMG signal equal to five times the standard deviation of this reference value was used to determine the premotor reaction time (Hopper et al., 1998).

Response time was defined as the total time between the presentation of the visual stimulus until the moment when the blow hit the target (punch ball) marked with reflective markers and showing a movement of 1 cm. Both response times and motor reaction times were measured, using the motion analysis system with a sampling frequency of 400 Hz. The technique used was the semicircular kick performed at the height of the head of the athlete, and the distance from the target was standardized, ensuring that the support foot, in the pre- and post-pace training moments, was placed in the same position demarcated on the floor.

#### Determination of Movement Time, Time of Performance, and Electromechanical Delay

The movement time was determined as the time interval between the response time and the kinematic motor reaction time, the performance time as the time interval between the premotor reaction time and the response time, and the electromechanical delay as the difference between the premotor reaction time and the kinematic response time.

#### Vertical Jump Test

Vertical jump performance was evaluated, using the CMJ test. All the athletes were familiar with the type of jump. The protocol consisted of three attempts, performed pre and post the pace training protocol and at each interval of the 2-min rounds. The test consists of adopting a standing position with the hands on the hips, bending the knees close to 90°, and performing a maximum jump. Performance (i.e., jump height) was measured, using a piezoelectric force platform (Kistler, Quattro jump, 9290AD, Winterthur, Switzerland), with a sampling frequency of 500 Hz. Three attempts were allowed, and the best jump was considered for the analysis. For the analysis of the height of the jump, the software of the force platform (Kistler, Quattro jump, 9290AD, Winterthur, Switzerland) was used.

### Statistical Analysis

Descriptive statistics (mean and standard deviation) were used to present the data. Normality was verified, using the *Shapiro–Wilk* test and homogeneity by the Levene test. ANOVA with repeated measures followed by the Tukey's test was used to compare the HRDP and HR of rounds 1, 2, and 3, as well as performance in the CMJ, and the *t-*test or Wilcoxon test for dependent samples was used to compare the motor variables of the roundhouse kick pre- and post-pace training. The magnitude of the differences was verified, using the effect size according to (*d*) (Cohen, 1969) or (*r*) (Pallant, 2007) and classified as trivial (<0.25), small (0.25–0.50), moderate (>0.50–1.0), or large effects (>1.0) (Rhea, 2004). A significance level of p < 0.05 was adopted. For the analysis and treatment of the data, Microsoft Office Excel 2007, Matlab 7.1 (MathWorks, Natick, MA, USA), and SPSS 17.0 software (SPSS Inc., Chicago, IL, USA) were used.

## Results

Table 1 presents the mean and standard deviation of the variables of the specific taekwondo test in taekwondo athletes.

**Table 1:** Mean and standard deviation of the variables obtained during the progressive specific taekwondo test (PSTT) (*n* = 12).

**KF_**MAX**_**	**KF_**AT**_ (pace)**	**HR_**MAX**_**	**HRDP**
33 ± 4 kicks	18 ± 2 kicks	195 ± 7 bpm	179 ± 8 bpm

*KF_MAX_, maximal kick frequency; KF_AT_, anaerobic threshold kick frequency; HR_MAX_, heart rate maximal; HRDP, heart rate deflection point*.

Table 2 shows the internal load responses with the HR values obtained in the PSTT and during the three rounds of pace training performed at the intensity corresponding to KF_AT_. During round 1, a lower internal load value was observed compared with HRDP (*p* = 0.0001). No differences were observed concerning an internal load and HRDP to rounds 2 (*p* = 0.456) and 3 (*p* = 1). The difference verified, using the effect size, presented a large effect (*d* = 1.360), comparing HRDP and HR during round 1. During rounds 2 and 3, the differences verified, using the effect size, presented trivial effect, comparing HRDP and HR (*d* = 0.167 and *d* = 0, respectively).

**Table 2:** Mean and standard deviation of the variables obtained during the pace training at an intensity corresponding to the intensity of the heart rate deflection point (HRDP) (*n* = 12).

**HRDP (CI)**	**HR_**ROUND1**_ (CI)**	**HR_**ROUND2**_ (CI)**	**HR_**ROUND3**_ (CI)**
179 ± 8 bpm (174 – 185)	167 ± 10 bpm^a^ (160 – 173)	178 ± 11 bpm^b^ (171 – 184)	179 ± 10 bpm^b^ (173 – 186)

*HR, heart rate; internal load value for rounds 1, 2, and 3 in comparison with the HRDP obtained for the pace identified during the PSTT*.

a *Different to HRDP*.

b *Different to round 1*.

*CI, confidence interval*.

Table 3 shows the performance in the CMJ. No difference in mean values pre- and post-pace training was observed (*p* = 0.227). The difference in the performance of CMJ post compared with the values was observed for performance in the intervals of rounds 1 (*p* = 0.011) and 2 (*p* = 0.028). However, a small effect size was observed compared with pre and post CMJ (*d* = 0.264) and performance post compared with values in the intervals of rounds 1 (*d* = 0.422) and 2 (*d* = 0.388).

**Table 3:** Mean and standard deviation of the performance in the countermovement jump test (CMJ) pre, post, and in the interval of the rounds of pace training performed at the intensity corresponding to the kick frequency at the anaerobic threshold (*n* = 12).

**CMJ_**PRE**_ (CI)**	**CMJ_**REST1**_ (CI)**	**CMJ_**REST2**_ (CI)**	**CMJ_**POS**_ (CI)**
46.4 ± 5.33 cm (42.9 – 50.0)	45.4 ± 6.01 cm (41.4 – 49.4)	45.7 ± 5.67 cm (41.9 – 49.4)	47.9 ± 5.71 cm^*^ (44.1 – 51.7)

*CMJ_PRE_, value for CMJ pre-pace training; CMJ_REST1_, value for CMJ during rest between rounds 1 and 2; CMJ_REST2_, value for CMJ during rest between rounds 2 and 3; CMJ_POS_, value for CMJ post-pace training*.

* *difference between rests 1 and 2 to post; CI, confidence interval*.

Table 4 presents the mean and standard deviation of the parameters associated with the neuromuscular and motor performance of the roundhouse kick during the evaluation pre and post the training of pace rounds in taekwondo athletes. No differences (*p* > 0.05) were observed between the moments pre- and post-pace training for CMJ and any of the variables associated with the reaction performance and motor response of the roundhouse kick, but a significant increase in the height of the vertical jump was observed in the CMJ test post-training in relation to the jumps recorded in the intervals between rounds. The differences verified using the effect size presented trivial and small magnitude effect.

**Table 4:** Mean and standard deviation and the effect size (ES) of the parameters associated with the neuromuscular and motor performance of the roundhouse kick during the evaluation pre and post the training of pace rounds in taekwondo athletes (*n* = 12).

	**Pre**	**Post**	**(CI)**	***P***	***d* (ES)**	***r* (ES)**
Premotor reaction time (ms)	311 ± 153	295 ± 76	(214 – 409)	0.548	−0.141	–
Response time (ms)	843 ± 235	743 ± 103	(677 – 999)	0.272	–	0.037
Motor reaction time kinematic (ms)	420 ± 147	404 ± 88	(327 – 514)	0.570	−0.139	–
Movement time (ms)	423 ± 231	339 ± 98	(276 – 569)	0.195	–	0.040
Electromechanical delay (ms)	109 ± 50	109 ± 51	(77 – 142)	0.993	−0.002	–
Performance time (ms)	532 ± 258	448 ± 96	(368 – 696)	0.239	–	0.056
Performance CMJ (cm)	46.4 ± 5.3	47.9 ± 5.7	(42.9 – 51.7)	0.130	0.260	–

*CMJ, countermovement jump test*.

## Discussion

The present study aimed to verify the effects of a pace training session at an intensity corresponding to KF_AT_ on the internal load response (HR), performance of the CMJ test, and parameters associated with the premotor reaction time, motor reaction time, and motor response of roundhouse kicks in taekwondo athletes. The observed results confirmed the hypothesis formulated for the present study, in which we suggest that the internal load (HR) observed during a pace training session would be similar to the intensity corresponding to that observed for pace intensity identified during a specific taekwondo test. On the other hand, the hypothesis that pace training would cause a reduction in the performance of the variables associated with neuromuscular performance and CMJ, as well as in the roundhouse kick reaction and response time variables in taekwondo athletes, was rejected.

In taekwondo, when planning a training program, it is necessary to consider the specific demands of the sport and its intermittent characteristics (Matsushigue et al., 2009; Campos et al., 2012; Del Vecchio et al., 2016). It is also necessary to take into account the principle of individuality, from the parameters of specific tests, such as the one used in the current study (PSTT) to help identify the internal load responses and prepare a more assertive intervention, using the individual pace in order to obtain greater control of the specific E:P ratio of the athlete and the expected adaptations from the intervention.

In the present study, the anaerobic threshold pace of the athletes, characterized by the KF_AT_, and with a mean E:P ratio of ~1:4, generated internal load (HR) responses (167, 178, and 179 bpm, for the rounds 1, 2, and 3, respectively). Only in the first round was observed an internal load value lower than that of the HRDP determined during the PSTT (*p* = 0.035). In addition, it was not possible to verify a negative influence (*p* > 0.05) of the pace intensity (Table 2) on the parameters associated with the neuromuscular and motor performance of the roundhouse kick in taekwondo athletes.

The movement time, both pre and post, observed in the present study presented better performance (shorter times) in relation to the athletes of the Spanish team and with higher values and worse performances in relation to the athletes of the German team (Hermann et al., 2008; Falco et al., 2011). The pre-pace training response time values observed in the present study were higher than those observed in a previous study, while the post-pace training values were lower (Sant' Ana et al., 2017). When comparing the response time values with the values (646 ms) observed in the athletes of the German team (Hermann et al., 2008), the athletes in this study presented higher values, and the values after the pace protocol were similar to the values (740 ms) observed in athletes from the Spanish team (Falco et al., 2011). Several factors may have influenced the values and differences observed between the present study and those mentioned above: (1) the intensity applied during the pace protocol, (2) the level of the athletes, and (3) the height of the kicking target. In the present study, the target was positioned at the height of the head of the athlete, while, in the study by Sant' Ana et al. (2017), the target was placed at the height of the trunk, in the study with athletes of the German team (Hermann et al., 2008) at the height of the waist, and the study with athletes of the Spain team (Falco et al., 2011) at the height of the trunk. Additionally, the measurement instruments used to determine the variables may have influenced the observed differences. In the studies by Hermann et al. (2008) and Falco et al. (2011), the variables time of movement and response time were determined by means of kinematics, like in the present study; however, the sampling frequency used to record the movement of the kick in both studies (150 and 300 Hz, respectively) differs from that used in the present study (400 Hz). In the study by Sant' Ana et al. (2017), the authors determined the response time, using accelerometers, making comparisons difficult.

The premotor reaction time values of the present study, both pre- and post-pace protocol, are higher than those observed in a previous study with seven taekwondo athletes (Sant' Ana et al., 2017). On the other hand, when observing the effect of pace training on the intensity, corresponding to the maximum oxygen consumption in taekwondo athletes, worsening of the premotor reaction time of the athletes was observed, as well as a decrease in the impact of the roundhouse kick (Sant' Ana et al., 2017). Associated with this, the fact that no changes in the electromechanical delay were observed allows us to infer that the pace protocol at the intensity corresponding to KF_AT_ did not compromise the neuromuscular performance of the roundhouse kick and did not generate fatigue in the athletes. It should be noted that the electromechanical delay can be influenced in situations of fatigue, being responsive to processes, such as propagation of the action potential; an excitation-contraction coupling process; triggering system of gamma motor neurons; and the recruitment of muscle fibers, factors that influence the time between the start of muscle activation and limb acceleration (Norman and Komi, 1979).

Another important finding of the present study that reinforces that the intensity of the KF_AT_ does not seem to compromise the performance and/or generate fatigue in the athletes is the fact that this intensity generated a potentiation effect in the performance of the CMJ (Table 3). After the three rounds of the pace protocol, there was a 3.1% improvement in the performance of the CMJ compared with pre, and a significant improvement of 5.4 and 4.8% compared with the CMJ post rounds 1 and 2, respectively. A case study with a World Karate Champion athlete (Loturco et al., 2017) also observed the improvement effect (2.9%) in the performance of the CMJ test pre and post a simulated fight. The authors of this study suggest that, in karate, there is little demand for kicking and lower limb techniques, as reported by Chaabene et al. (2014), and, as a consequence, a post-activation potentiation effect in CMJ was observed.

However, this fact goes against the findings of the present study, in which the kick technique was required throughout the pace training, and we also observed a post-activation potentiation effect on the jumping performance. Therefore, our findings demonstrate that the post-activation potentiation in the performance of the jump after simulated rounds and or fights may be associated with the intensity and economy capacity of the athlete according to the demand. As observed in the present study, when the athlete performs motor actions at a relative pace that does not overlap the KF_AT_, this should not impair the performance of the specific kick technique in any of the components associated with the reaction and response time of the kick. The post-activation potentiation observed in the CMJ may also be the result of aspects of the mechanics and kinetics involved in the CMJ jump performance test. The jump is a motor gesture distinct from those performed by combat sports athletes. In the execution of the roundhouse kick, there is flexion in the hip joint and extension of the knee joint, while, in the jump, there is a hip extension and knee extension. Thus, some of the mechanisms suggested by Baker (2003), such as increased recruitment of motor units, improved synchrony of the firing of nerve impulses, and, mainly, the reduction of the influence of central inhibitory mechanisms (Renshaw cells) and peripherals (Golgi tendon organ) and increased reciprocal inhibition of the antagonistic musculature could better explain the post-activation potentiation effect observed in CMJ, after a simulated fight or pace training.

Thus, the results of the present study demonstrate that the incremental evaluation in taekwondo athletes can be an alternative tool to establish the individualized E:P ratio. HR can be used as an internal load marker and, through HRDP and KF_AT_, can be used as an external load indicator associated with the anaerobic threshold, as well as to establish the individual pace (E:P) in dynamics with motor actions close to those performed by athletes in combat to propose pace training rounds, to generate expected physiological responses, and to obtain greater control of the intensity and organic adaptations that are desired individually for each athlete. Additionally, no difference was found between the HRDP identified in an incremental test on a treadmill and the PSTT (Sant' Ana et al., 2019). Just as there was no difference between the anaerobic threshold determined in a rectangular load protocol, by means of fixed concentrations of 4 mmol·L^−1^ (Heck et al., 1985), compared with the anaerobic threshold determined by the HRDP when applying the PSTT (Sant' Ana et al., 2009). All of these findings point to the possibility of this variable being used to control the internal load of athletes and the marker of internal response for a given external load applied.

Finally, as in taekwondo, there is predominance of aerobic demand brought by determinant actions of high intensity, which make demands on the alactic anaerobic system (Campos et al., 2012), and which denote the modality; this characteristic is imposed by the relationship of high and low-intensity activities. Considering the KF_AT_, estimated by the HRDP, it may be a possibility for the training of athletes of the modality, mainly when greater control and precision are required in relation to the internal load that it is intended to impose on the athlete. In addition, the use of pace training protocols enables the development of specific skills (kicking techniques), including the determinant anaerobic components, since high-intensity kicks, characteristic of the intermittent mode, are present throughout the incremental protocol and during the pace training protocols.

This study had some major limitations that should be considered while interpreting our findings. The first one is the small sample size and the level of the athletes assessed. Thus, future studies should include a larger number of athletes with different competitive levels. In addition, only the intensity corresponding to the HRDP was evaluated. Thus, future studies should compare different relative paces above and below the HRDP. Lastly, they may include other internal load variables, such as oxygen uptake and blood lactate concentration, are necessary to better understand the effects of pace on internal load and neuromuscular parameters and about kick response time in taekwondo athletes. Furthermore, it is important to emphasize the aspects related to the nature of the incremental test (i.e., PSTT) and pace protocols with the intrinsic characteristics of combat sports. Among these aspects is the inability to maintain and reproduce a similar intensity, speed, and strength of the kicks during the test. Therefore, future studies with the use of electronic vests may be an alternative to ensure that the minimum required intensity is reached during the execution of the kicks.

## Practical Applications

The pace training, using the KF_AT_ parameter estimated by the HRDP, is a simple methodology to execute, presents low cost, and provides relevant sport-specific information. Outcomes from this study type appoint to practical applications of the methodology with a great advantage for coaches and athletes to not only evaluate performance and determine the optimal workload but also to organize and monitor taekwondo training programs, especially those focused on improving the aerobic component of the sport. The protocol proposed in the present study allows obtaining relevant and specific information on aerobic capacity and power (i.e., maximal kick frequency, KF_AT_, and pace) simpler and cheaper for coaches and athletes. Finally, we believe that our findings are important to encourage coaches and trainers to raise training loads monitoring and a prescription with greater control and precision in relation to the internal load that it is intended to impose on the athlete.

## Conclusions

The internal load (HR) during rounds of pace training performed at the intensity corresponding to KF_AT_ is equivalent to the anaerobic threshold (HRDP) and can be considered in the prescription of training when the aim is to control the internal load responses without inducing fatigue since no impairment in neuromuscular performance was observed in the performance of the CMJ and the roundhouse kick technique.

## Data Availability Statement

The raw data supporting the conclusions of this article will be made available by the authors, without undue reservation.

## Ethics Statement

Ethical approval was obtained from the local Human Research Ethics Committee of the Federal University of Santa Catarina (protocol number 145882). The patients/participants provided their written informed consent to participate in this study.

## Author Contributions

JS and RS carried out the data collection. JS carried out all the statistical analyses. All authors conceived the study design, participated in the interpretation of data, drafted the manuscript, and read and approved the final version of the manuscript.

## Conflict of Interest

The authors declare that the research was conducted in the absence of any commercial or financial relationships that could be construed as a potential conflict of interest.

## Publisher's Note

All claims expressed in this article are solely those of the authors and do not necessarily represent those of their affiliated organizations, or those of the publisher, the editors and the reviewers. Any product that may be evaluated in this article, or claim that may be made by its manufacturer, is not guaranteed or endorsed by the publisher.
